# Indocyanine green fluorescence kinetics during hypothermic oxygenated machine perfusion might predict graft function after liver transplantation

**DOI:** 10.1007/s00423-026-04158-4

**Published:** 2026-07-22

**Authors:** Mareike Franz, Jörg Arend, Antonia Bollensdorf, Frederike Stelter, Ahmed Sanin, Thomas Wartmann, Ulf D. Kahlert, Verena Keitel-Anselmino, Theresa Florian, Roland Croner

**Affiliations:** 1 Department of general-, visceral-, vascular- and transplant surgery, Leipiziger Str. 44, Magdeburg, 39120 Germany; 2 Department of Molecular & Experimental Surgery (MES), Department of general-, visceral-, vascular- and transplant surgery, Leipiziger Str. 44, Magdeburg, 39120 Germany; 3 Department of gastroenterology, hepatology and infectiology, Leipiziger Str. 44, Magdeburg, 39120 Germany

**Keywords:** Donor liver viability, Extended- criteria donor grafts, Graft assessment, Perfusate fluorescence intensity, Hepatocellular uptake, Hypothermic oxygenated perfusion, Early graft dysfunction prediction, Hepatocyte transporter function, Mitochondrial stress indicators, Predictive threshold for graft failure

## Abstract

**Background:**

This study evaluates whether indocyanine green (ICG) fluorescence during hypothermic oxygenated machine perfusion (HOPE) can predict early allograft dysfunction (EAD) after liver transplantation.

**Methods:**

Seventeen donor livers underwent HOPE before transplantation. After one hour, ICG was administered via the portal line. Perfusate samples were collected every five minutes for 50 min; liver tissue and bile samples were obtained during perfusion and after reperfusion. Fluorescence intensity (FI) was quantified using standardized microplate analysis. Associations with donor characteristics and clinical outcomes were analyzed.

**Results:**

EAD occurred in 29.4% of recipients and primary non-function in 5.9%. Perfusate FI differed significantly between EAD and non-EAD grafts at 40 and 45 min (*p* = 0.006; *p* = 0.05). FI in post-reperfusion liver biopsies was higher in dysfunctional grafts (*p* = 0.01). Bile fluorescence showed no predictive value. ROC analysis demonstrated strong discrimination for perfusate samples at 40 and 45 min (AUC 0.875; 0.889) and for post-reperfusion tissue (AUC 0.886). Donor variables did not correlate with ICG kinetics.

**Conclusion:**

In this exploratory pilot study, perfusate fluorescence measurements during HOPE were associated with EAD. These findings suggest that fluorescence- based assessment during machine perfusion may provide functional information on graft quality.

## Introduction

Liver transplantation, first clinically performed by T. Starzl in the 1960 s, remains the only curative treatment for patients with terminal liver diseases. However, the shortage of suitable donor organs has led to an increasing reliance on extended criteria donor (ECD) livers, which are more vulnerable to ischemia-reperfusion injury (IRI). Given that donor organ quality significantly impacts post-transplant function and long-term survival, accurate risk assessment is essential. In the Eurotransplant region, ECD livers now constitute over 50% of transplants, and this proportion is expected to rise further. Despite expanding the donor pool, only 70% of donor livers are ultimately transplanted [1, 2]. Machine perfusion (MP) has emerged as a promising strategy to enhance the preservation and viability assessment of donor livers before transplantation. Viability assessment of normothermic perfused grafts is already clinical routine based on different established parameters, such as lactate clearance, glucose metabolism, pH of the perfusate and biliary parameters [3, 4]. Hypothermic oxygenated machine perfusion (HOPE) in particular, has gained widespread clinical acceptance due to its simplicity and feasibility in clinical settings and supported by convincing data [5–7]. However, currently there is no standardized viability assessment during HOPE. Schlegel et al. demonstrated that flavin mononucleotide (FMN), released during mitochondrial stress, can serve as a viability marker for hypothermic perfused livers and predict Early Allograft Dysfunction (EAD) and one- year patient´s survival [8]. However, due to cost and technical challenges, this method is not yet feasible for routine clinical use. Rauter et al. evaluated Syndecan-1 as marker of endothelial dysfunction and therefore parameter for viability assessment in the hypothermic machine perfusion and showed that EAD can be predicted [9]. Despite promising data there is no viability assessment for the clinical routine yet. Current clinical parameters such as the Model for End-Stage Liver Disease (MELD) score and donor-specific risk factors, including cold ischemia time (CIT), donor age, and transaminase levels offer limited insight into real-time hepatocellular function. Indocyanine green (ICG) is a fluorescent dye that has been used clinically since 1954 following FDA approval [10, 11]. Its absorption spectrum lies within the near-infrared (NIR) range. When stimulated with light at a wavelength of approximately 840 nm, the maximum fluorescence emission occurs when protein-bound. Following intravenous injection, ICG is taken up by hepatocytes via organic anion-transporting polypeptides (OATP) and sodium taurocholate co-transporting polypeptides (NTCP), with a plasma half-life of approximately 150–180 s. After about 10 min, ICG is excreted unconjugated via the multidrug resistance-associated protein (MRP2) into bile [10–12]. Since ICG is not subject to enterohepatic circulation, it serves as a dynamic and reliable tool for assessing liver function. The retention rate at 15 min (ICG R-15) can provide insight into underlying liver function. In intensive care medicine, a significant correlation between patient survival and the plasma disappearance rate (PDR) of ICG has been demonstrated [13]. This has led to its adoption in hepatobiliary surgery. A critical aspect of liver function assessment is the ability of hepatocytes to efficiently uptake and excrete ICG, making it a valuable biomarker for hepatic function and an intraoperative diagnostic tool. Approaches to use ICG R-15 to predict graft quality and prognosis beforehand in the donor or after liver transplantation in the recipient have been described [14–16]. Several articles describe the use of ICG for graft assessment in normothermic machine perfusion (NMP) [17]. But still there is no standardized viability assessment of donor livers during HOPE before transplantation.

The aim of this study was to identify a threshold of the ICG clearance in donor livers during HOPE as predictor for post-transplant liver function. It is the first clinical pilot study (DRKS00033469) that aims to investigate the relationship between the dynamics of ICG in donor livers during HOPE. The findings may contribute to improving donor organ selection, reducing post-transplant complications, and optimizing patient outcomes.

## Materials and methods

### Patients and inclusion criteria

Patients with terminal liver diseases or hepatocellular carcinoma (HCC) who underwent liver transplantation from 12/2023 to 02/2025 were included in the study after written consent. Inclusion criteria were: Patient age ≥ 18 years, terminal liver disease as indication for transplantation, tumor diseases as indication for transplantation, Eurotransplant listing (ET), preoperative hypothermic machine perfusion of the donor liver graft. Patients were excluded if: age < 18 years, pregnancy, breastfeeding, acute liver failure (HU criteria), chronic renal insufficiency (GFR < 45 ml/min), iodine allergy, hyperthyroidism, known allergy to ICG. The included patients underwent the regular evaluation process for liver transplantation in our transplant center after multidisciplinary transplant board recommendation. This study was approved by the institutional ethics committee ([151/23]), and all procedures were conducted in accordance with the Declaration of Helsinki. This study is registered in the German Clinical Trials Register (DRKS) under the identifier DRKS00033469.

Key donor characteristics, including donor age, BMI, CIT, and general bloodwork, were recorded. Post-transplant outcomes, including early allograft dysfunction (EAD) defined by Olthoff et al. [18] were documented and analysed. Postoperative complications were classified according to the Clavien-Dindo (CD) classification system [19].

### Indication for donor liver graft hypothermic perfusion

The decision for hypothermic machine perfusion depended on specific risk factors of the donor liver graft. These were defined as: cold ischemia time, steatosis hepatis > 30%, cardio pulmonal reanimation for cardiac arrest of the donor.

### Hypothermic liver graft perfusion (HOPE)

**Fig. 1:**
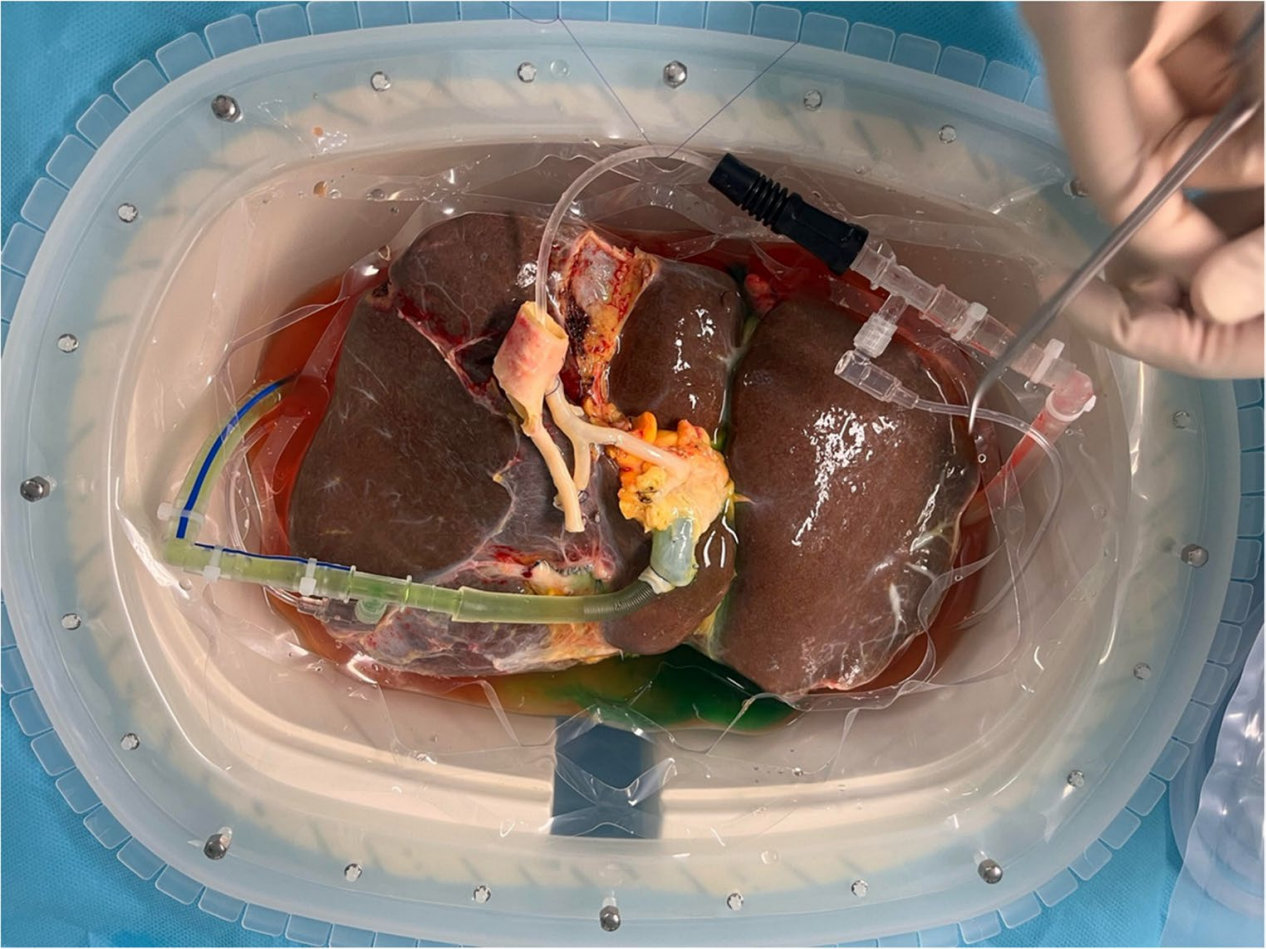
Indocyanine green (ICG) inflow via portal vein, outflow via inferior vena cava (IVC) and recirculation of the perfusate during hypothermic organ perfusion of the donor liver

Machine perfusion was performed with the Liver Assist (XVIVO, Mölndal, Sweden). Donor livers were stored cold in Histidine- Tryptophane- Ketoglutarate (HTK) Custodiol ^®^ solution (Dr. Franz Köhler Chemie GmBH, Alsbach- Hähnlein, Germany) until the arrival at our center. The livers were prepared backtable for the vascular connection to the machine and subsequent anastomoses. 2 L of solution for machine perfusion the Belzer UW ^®^ Cold Storage Solution (University of Wisconsin Solution, Madison, WI, USA) were used per perfusion. Dual (arterial and portal) Hypothermic oxygenated perfusion (DHOPE) at 11–12 °C was performed. The portal vein and hepatic artery were cannulated and connected to the XVIVO perfusion system, either via an aortic patch or through the celiac trunk. Arterial pressure was maintained between 20 and 30 mmHg, while portal venous pressure was set at 3–5 mmHg. Flow rates were continuously monitored, with acceptable ranges defined as 0.1–0.25 L/min for the portal vein and 80–120 mL/min for the hepatic artery. Perfusion pressures were adjusted accordingly in response to deviations in flow, being increased when flow was insufficient and decreased when excessive. Blood gas analysis was performed to assess oxygenation of the perfusate, ensuring a partial pressure of oxygen (pO₂) above 600 mmHg, as recommended by the manufacturer (Fig. 1).

### Study design and sample harvesting

**Fig. 2:**
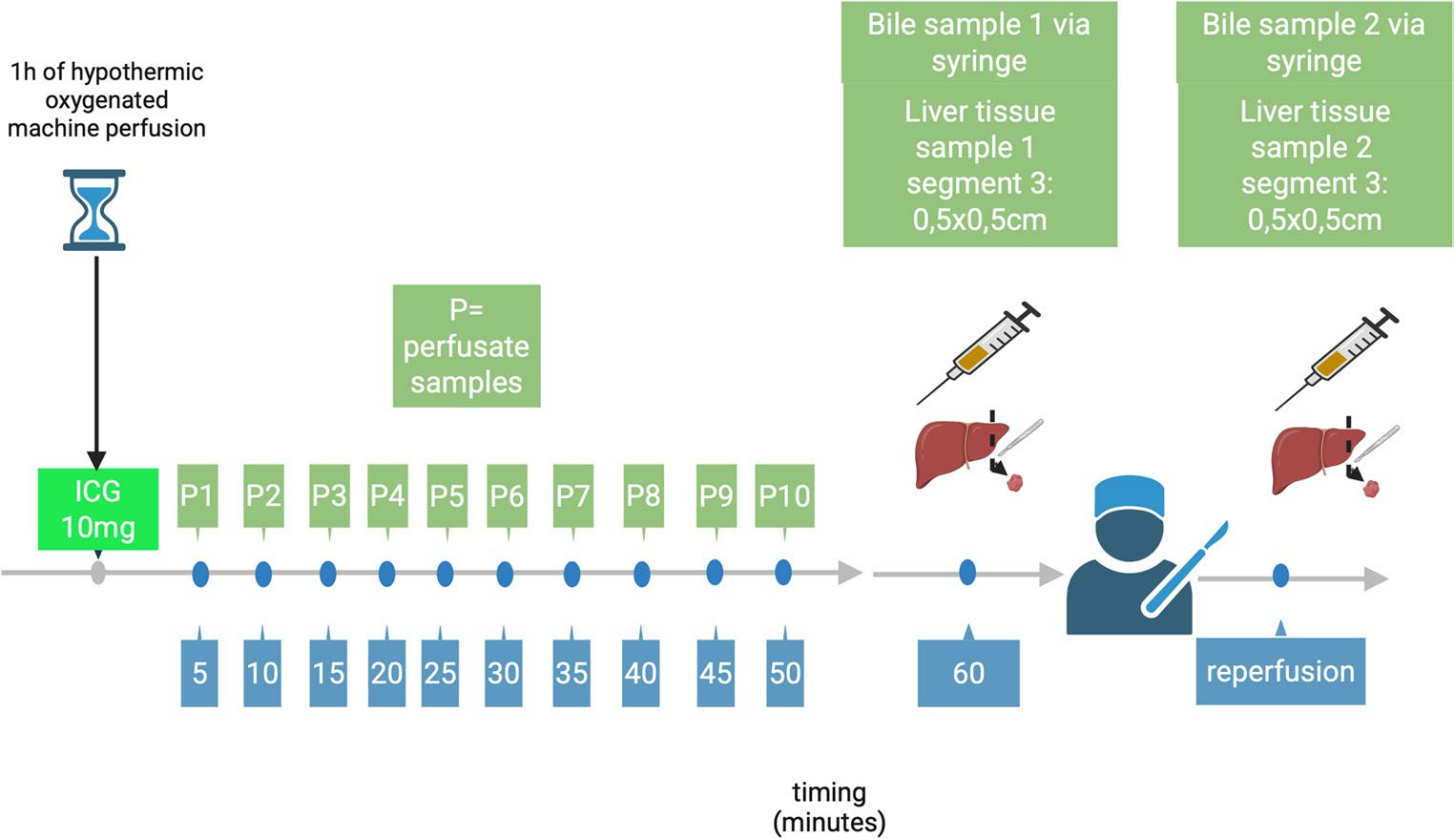
Indocyanine green (ICG) application, perfusate, bile and tissue sampling during hypothermic liver graft perfusion (HOPE) and after reperfusion created with Biorender.com

A native sample of the perfusate (2 ml) was harvested as control at the beginning of the perfusion process. After at least one hour of machine perfusion 10 mg of ICG (Verdye ^®^ 25 mg) were dissolved in Aqua ad injectionem 5 ml. Two ml of this solution were injected into the portal vein site (sample line). Afterwards the sample line was flushed with sterile NaCl 0.9% 10 ml. Every 5 min 2 ml samples of the perfusate were harvested up to 50 min after ICG injection. Sixty minutes after ICG injection a tissue sample 0.5 × 0.5xcm of the hypothermic perfused liver was taken from liver segment 3 under sterile conditions (liver 1). A small amount (1 ml) of bile (bile 1) was sampled with a 2 ml syringe at the same time. If there was no bile secretion at that time, the sample was taken at a later time point during machine perfusion (Fig. 2).

After reperfusion of the transplanted liver in the recipient, a second 0.5 × 0.5 cm sample of liver segment 3 was harvested (liver 2). Before the bile duct anastomosis a second bile sample with a 2 ml syringe was taken (bile 2). Following sample collection, specimens were placed in specimen containers and stored at 4 °C until further analysis. Tissue samples were sectioned into two thin slices (approximately 1.00–1.10 mm thick) and carefully positioned onto the quartz lenses of a Tecan NanoQuant Plate™. Fluorescence measurements were performed using a Tecan Spark 20 M microplate reader, with an excitation wavelength of 780 nm and an emission wavelength of 820 nm. To ensure measurement consistency and statistical robustness, the tissue sections were rearranged and remeasured twice under identical conditions. For liquid samples (perfusate and bile), triplicate aliquots were pipetted into a Brand 96-well microtitration plate (Cat. Nr. 781610), and ICG fluorescence intensity was measured using the same spectral configuration as for tissue samples. Acquired data were analysed and plotted using GraphPad Prism 10.

### Statistical analysis

The statistical analysis was performed using SPSS for Mac (Version 29.0.2.0(20) IBM Corporation Armonk, New York, USA). Descriptive statistics, including means, standard deviations, and interquartile ranges, were calculated for all relevant variables. To assess relationships between fluorescence intensity and donor characteristics, Pearson or Spearman correlation coefficients were used depending on data distribution. Differences between groups (e.g., EAD vs. non-EAD) were analyzed using independent t-tests or Mann-Whitney U tests. Receiver operating characteristic (ROC) analysis was used to determine fluorescence-based thresholds indicative of graft dysfunction. A p- value of < 0.05 was considered significant. Statistical figures were created using SPSS. Given the exploratory nature of this pilot study and the limited sample size, statistical analyses were primarily descriptive and hypothesis- generating.

## Results

### Patient characteristics

The mean age of the 17 recipients was 57.18 (SD 7.76) years. Of these 14 (82.4%) were male and 3 (17.6%) were female patients (Table 1). In 58.8% patients suffered from hepatocellular carcinoma (HCC). Of these patients 29.4% met the Milan criteria and 29.4% did not meet the Milan criteria. Reasons for liver cirrhosis was in 41.2% ethyltoxic liver cirrhosis and in 29.4% had MASLD cirrhosis. Other reasons for liver cirrhosis were viral hepatitis in 11.8% and in 17.6% other reasons such as cryptogenic cirrhosis, primary sclerosing cholangitis (PSC) or autoimmune hepatitis (AIH). In 11.8% recipients were scored as American Society of Anesthesiologists (ASA) Score 2, in 64.7% ASA 3, in 17.6% ASA 4 and 5.9% were ASA 5 (Table 1).

**Table 1:** Patient demographics who underwent liver transplantation after hypothermic organ perfusion (HOPE) of donor livers. ASH: alcoholic steatohepatitis, MASLD: metabolic dysfunction- associated metabolic liver disease (*n* = 17)

Age (years) ± SD	57.2 ± 7.8
ASA score ± SD	3.2 ± 0.7
Sex (male/female)	14/3
HCC n (%)	10 (58.8)
ASH n (%)	7 (41.2)
MASLD n (%)	5 (29.4)
Virus Hepatitis n (%)	2 (11.8)

### Liver graft characteristics

The data included livers from donors with an average age of 63.53 years (SD 18.04, range: 17–86) and a mean BMI of 27.76 kg/m² (SD 4.85). In 64.7% the livers came from male donors and in 35.3% came from female donors (Table 2). Mean time of donor cardiopulmonary resuscitation (CPR) was 6.7 min (SD 15.3). In 73.3% donors had moderate atherosclerosis and in 26.7% no atherosclerosis was identified.

**Table 2:** Characteristics of donor livers which underwent hypothermic organ perfusion (HOPE) and ICG clearance evaluation during liver transplantation (*n* = 17)

	Value
Age (years) ± SD	63.5 ± 18.0
Sex (male/female)	11/6
BMI (kg/m²) ± SD	27.8 ± 4.9
Donor Resuscitation n (%)	3 (18)
Sodium (mmol/L) ± SD	144.5 ± 5.6
Arteriosclerosis n (%)	11 (73.3)
Bilirubin (mg/dL) ± SD	12.8 ± 7.6
Quick value (%) ± SD	95.1 ± 18.0
Steatosis (> 30%) n (%)	2 (13.3)

In 40% the procurement team initiated frozen section analysis of the liver parenchyma. In 13.3% we saw regular liver parenchyma, 13.3% of the donor livers had fatty degeneration of at least 30%. In 13.3% of the donor livers early stage portal fibrosis was diagnosed. In 41.2% we accepted primary offers from Eurotransplant (ET), in 47.1% we accepted offers in the extended allocation and in 11.8% we received donor livers via rescue allocation. The average cold ischemia time (CIT) was 393.64 min (111.94), the duration of the hypothermic oxygenated machine perfusion was excluded. The average time of machine perfusion was 318 min (SD 251.22, range 180–650) (Table 2).

### Liver transplantation and perioperative outcome

Mean length of surgery was 273 min (SD 69.09). Mean time on the intensive care unit (ICU) was 11 days (SD 15.15). The mean length of hospital stay was 32 days (SD 28.36). In 29.4% recipients had complications which required intervention (e.g. endoscopic, operative), 23.5% required intensive care treatment and 17.6% died in the postoperative course. In 5.9% we saw Primary Non Function (PNF) which led to re- transplantation and we saw Early Allograft Dysfunction (EAD) in 29.4% of the recipients (Table 4). A significant correlation could be observed between EAD and ICU stay (*p* = 0.045), as well as between EAD and complications Clavien-Dindo (CD) ≥ 3 (*p* = 0.019) (Table 3). No problems with the HOPE occurred in the cohort.

**Table 3:** Perioperative and postoperative outcome measures in patients who underwent liver transplantation of hypothermic perfused (HOPE) donor livers (*n* = 17). EAD: Early Allograft Dysfunction CD: Clavien Dindo Classification of Surgical Complications, Mortality: In-hospital mortality, PNF: Primary Non- Function, ICU: Intensive Care Unit, SD: standard deviation

	Value
Cold ischemia time (min) ± SD	393.6 ± 111.9
Warm ischemia time (min) ± SD	52.6 ± 83.9
Operative time (min) ± SD	272.5 ± 69.1
HOPE (min) ± SD	318 ± 251
Hospital stay mean; days ± SD	32 ± 28.4
EAD n (%)	5 (29.4)
PNF n (%)	1 (5.9)
ICU days ± SD	11 ± 15.15
Morbidity CD > 3 n (%)	5 (29.4)
Mortality n (%)	3 (17.6)

### Indocyanine green signaling and clinical outcome

**Fig. 3:**
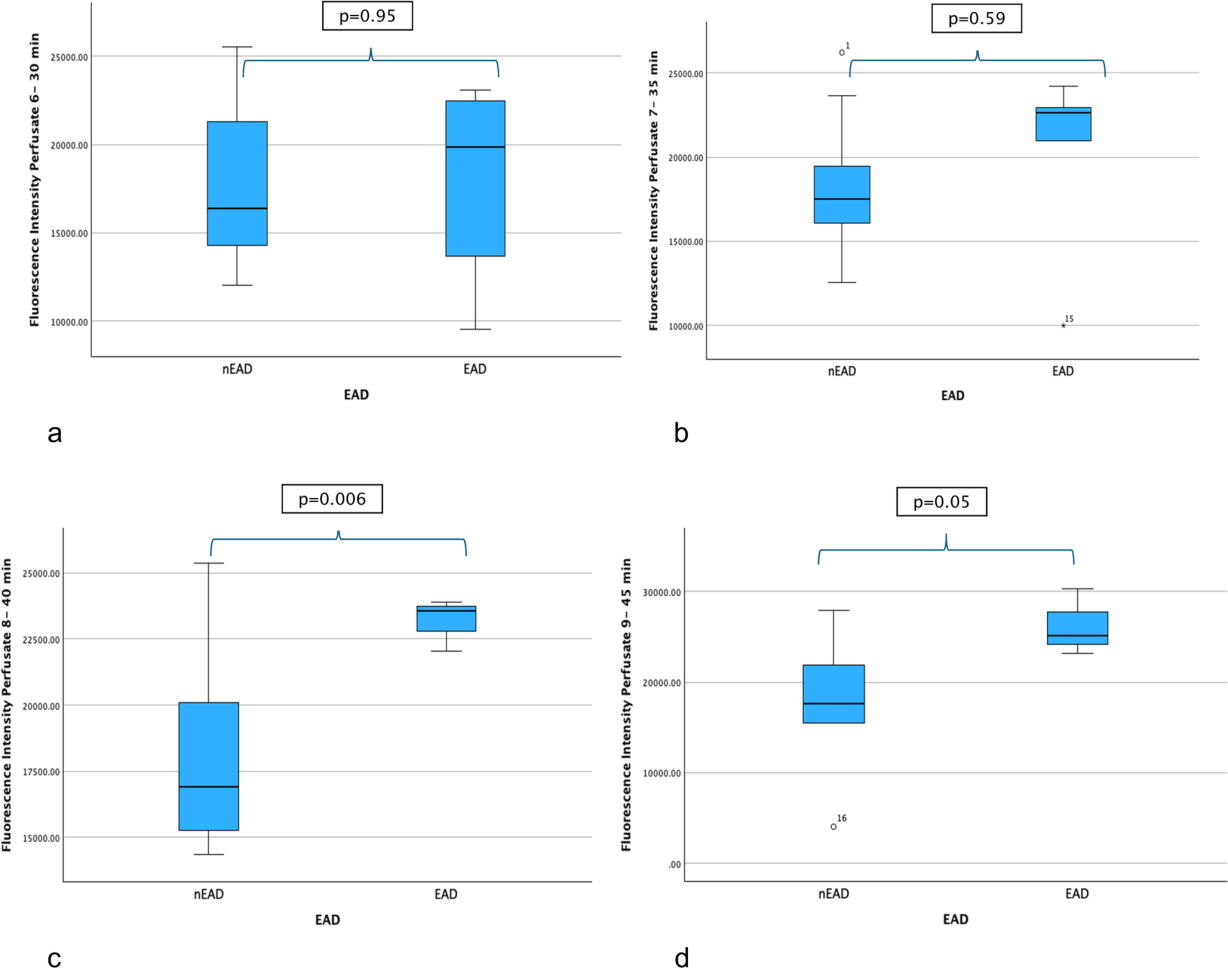
Fluorescence intensity of ICG in perfusate during hypothermic organ perfusion (HOPE) after (**a**) 30 min (P6), (**b**) 35min (P7), (**c**) 40 min (P8) and (**d**) 45 min (P9). EAD: Early Allograft Dysfunction; nEAD: no Early Allograft Dysfunction

**Fig. 4:**
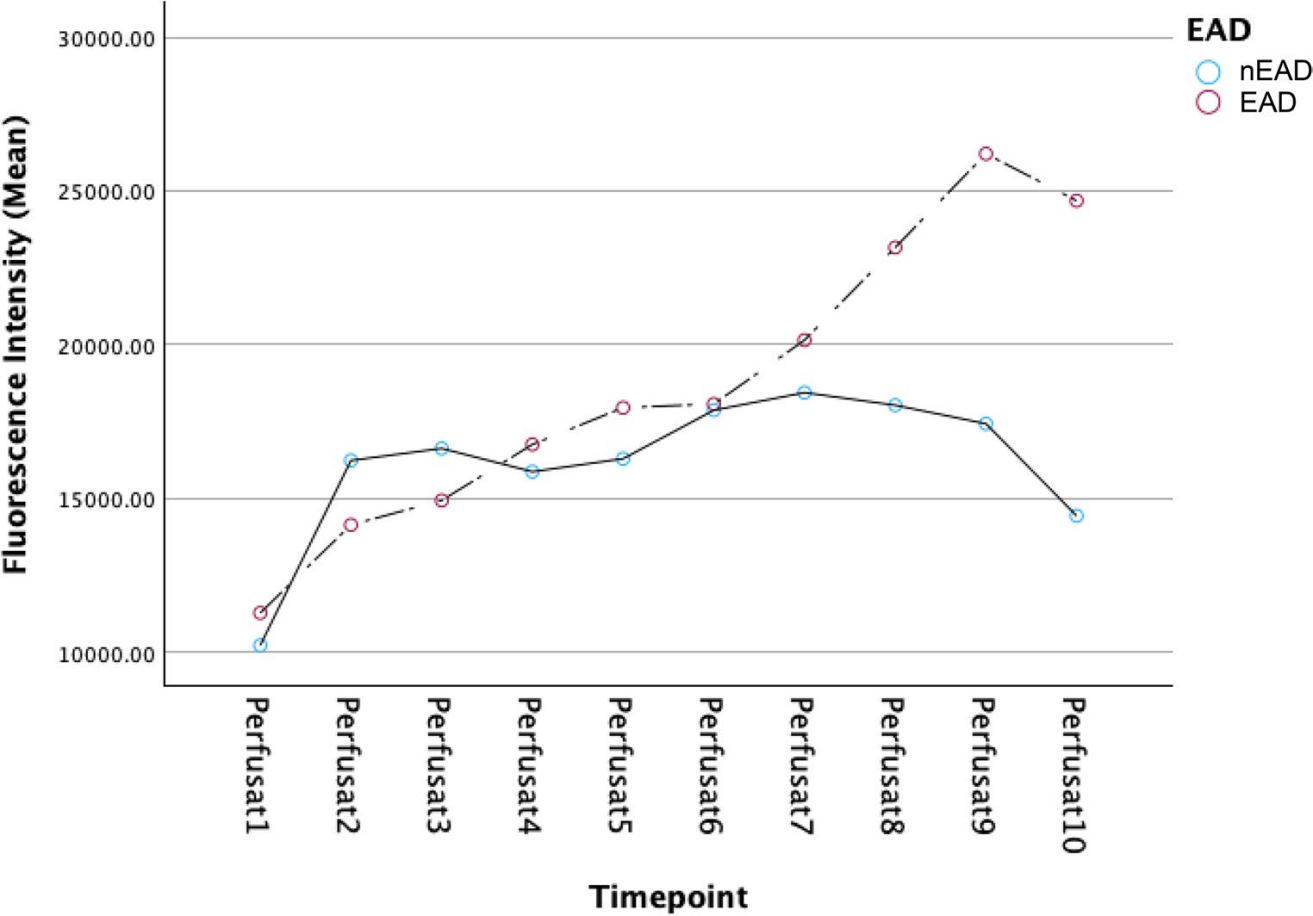
Fluorescence intensity of the perfusate during hypothermic organ perfusion (HOPE) of the donor livers on different timepoints of measurement, EAD: Early Allograft Dysfunction (red); nEAD: no Early Allograft Dysfunction (blue). P6 (30 minutes HOPE); P8 (40 minutes HOPE; EAD vs nEAD p=0.006); P9 (45 minutes HOPE; EAD vs nEAD p=0,05)

Mean fluorescence intensities in perfusate samples collected at defined time points after ICG injection were compared between grafts with EAD and no Early Allograft Dysfunction (nEAD). Significant differences in FI between EAD vs. nEAD were identified in perfusate 8 (*p* = 0.006) and perfusate 9 (*p* = 0.05), 40 and 45 min after ICG application during HOPE (Fig. 3). The FI increased over time in both groups, with a steeper rise observed in the EAD group beginning 30 min (perfusate 6) after ICG injection. In contrast, FI decreased in the nEAD group after this timepoint significantly (Fig. 4).

**Fig. 5:**
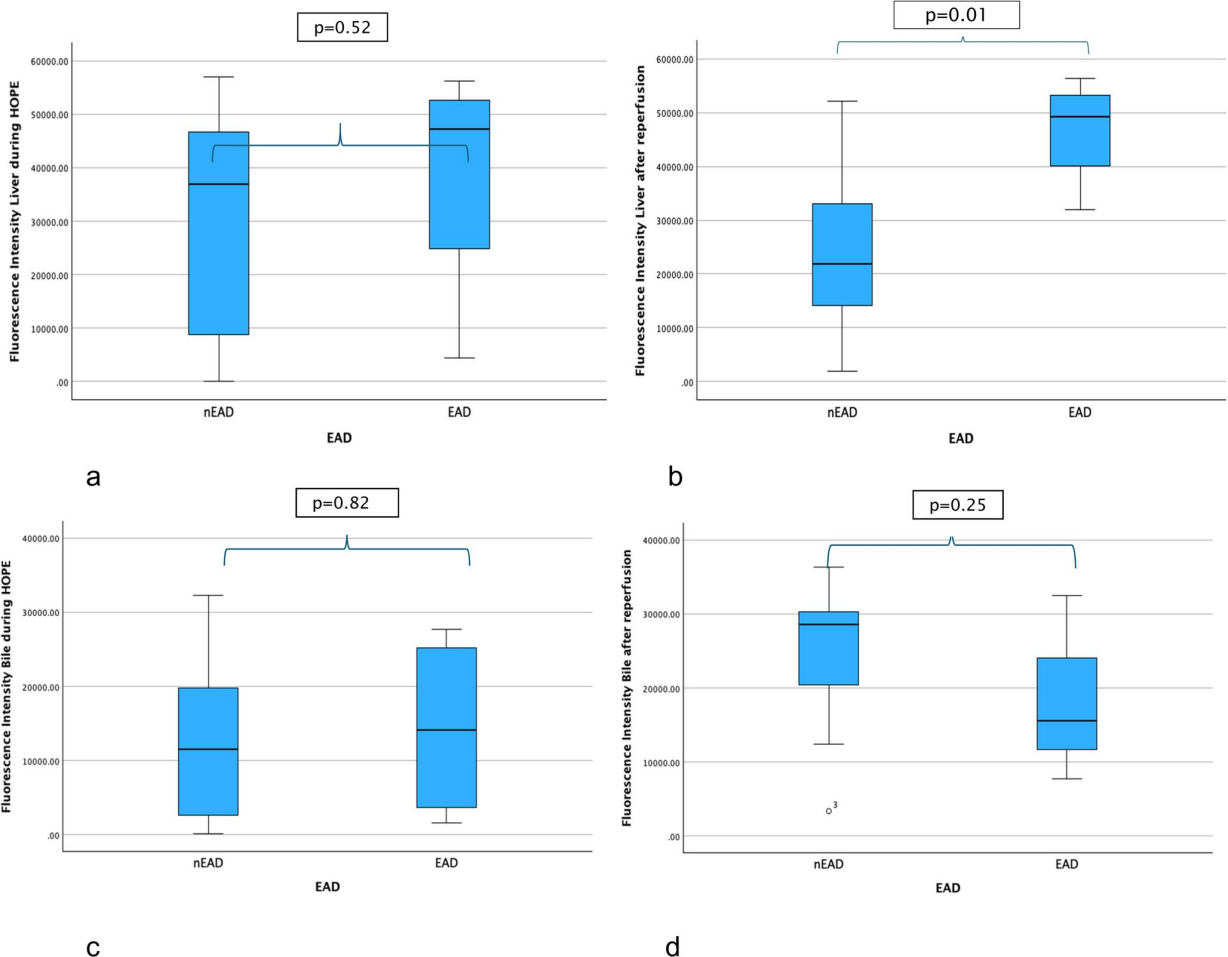
Fluorescence intensity of ICG in liver biopsies (**a**) after hypothermic organ perfusion (HOPE) and (**b**) after liver perfusion in the recipients; Fluorescence intensity of harvested bile (**b**) after HOPE and (**d**) liver perfusion in the recipients. EAD: Early Allograft Dysfunction; nEAD: no Early Allograft Dysfunction

The fluorescence intensity (FI) of the liver biopsies (liver 1) that developed EAD was slightly higher after 1 h HOPE than in the cohort who developed no early allograft dysfunction (nEAD) without statistical significance (*p* = 0.95). FI was significantly higher in the liver biopsy samples after reperfusion (liver 2) of the livers who developed an EAD vs. the livers who developed nEAD (*p* = 0.01) (Fig. 5). The FI of the bile sample during HOPE (bile 1) does not show significant difference between the EAD and nEAD group (*p* = 0.82). The bile sample after liver reperfusion (bile 2) showed no significant difference between EAD vs. nEAD (*p* = 0.25). However, a higher mean FI in the bile of nEAD livers after reperfusion can be identified (Fig. 5). Exploratory correlation analyses (Spearman) between the relevant ICG fluorescence timepoints and the individual laboratory components of EAD were performed. Perfusate fluorescence intensity at 40 and 45 min showed a positive correlation with AST levels on postoperative day 1 (> 2000IE/l) (*p* = 0.019, *p* = 0.004). Perfusate fluorescence at 40 min also showed a positive correlation with AST on postoperative day 5 (> 2000 IE/l) (*p* = 0.029). No other statistically significant correlations to individual components of EAD were observed.

**Fig. 6:**
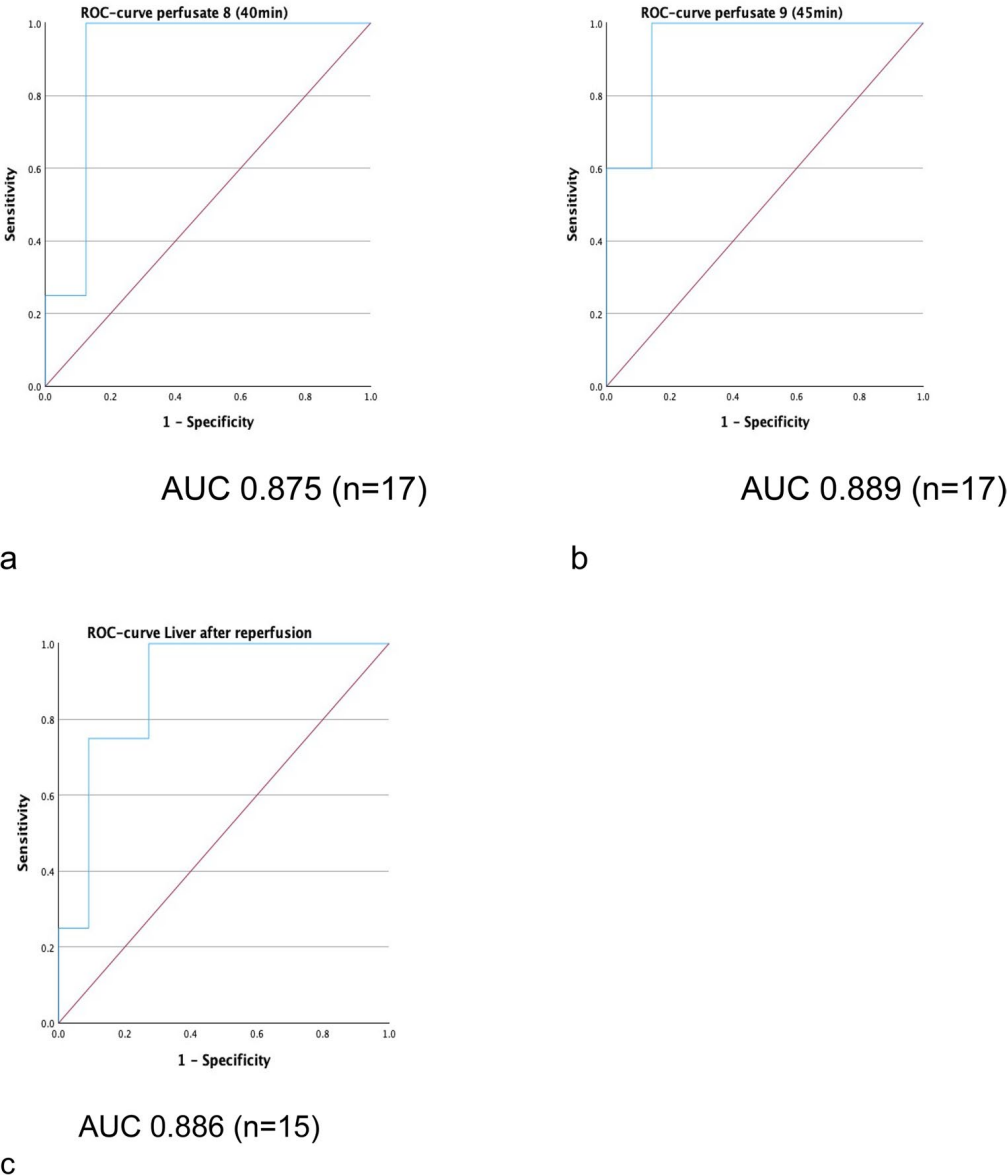
ROC curves: FI of the perfusate after (**a**) 40 minutes (P8), (**b**) 45 minutes (P9) during HOPE and ROC analysis of ICG fluorescence intensity in liver biopsy samples (**c**) after reperfusion as predictor for Early Allograft Dysfunction (EAD)

During ROC analysis between EAD vs. nEAD groups can be separated in perfusate samples 8, 9 and liver biopsy after reperfusion with strong predictive accuracy (AUC > 0.8) (Fig. 6). Due to missing values for perfusate 10 and the small group size a more detailed analysis of perfusate 10 was not performed.

In addition to ROC- AUC, the Youden Index (J= Sensitivity+ Specificity-1) was calculated to identify the optimal cut-off value for each marker (Tables 4 and 5).

**Table 4:** Area under the curve (AUC); ROC analysis of bile, liver biopsies and perfusate samples, showing the strong predictive value of liver 2 and perfusate after 40 (P8) and 45 min (P9)

Probe (*n*)	AUC (95% CI)
Bile 1 (14)	0.563 (95% CI 0.182–0.943)
Bile 2 (after reperfusion) (15)	0.364 (95% CI −0.43- 0.770)
Liver 1 (15)	0.659 (95% CI 0.350–0.968)
Liver 2 (after reperfusion) (15)	0.886 (95% CI 0.712–1.061)
Perfusate 4 (20 min) (17)	0.604 (95% CI 0.222–0.986)
Perfusate 5 (25 min) (17)	0.682 (95% CI 0.274–1.090)
Perfusate 6 (30 min) (17)	0.55 (95% CI 0.174–0.926)
Perfusate 7 (35 min) (17)	0.644 (95% CI 0.296–0.993)
Perfusate 8 (40 min) (17)	0.875 (95% CI 0.720–1.093)
Perfusate 9 (45 min) (17)	0.889 (95% CI 0.810–1.076)
Perfusate 10 (50 min) (12)	0.5 (95% CI −0.193- 1.193)

**Table 5:** Optimal cutoff values of fluorescence intensity (FI) of ICG in perfusate during hypothermic organ perfusion during various timepoints and FI of ICG in harvested bile and liver biopsies for early allograft dysfunction (EAD) Prediction (Youden- Index: Sensitivity + Specificity-1); ^*^: this parameter could not be evaluated due to incomplete data

	Optimal Cutoff (FI)	Youden Index
Perfusate 6 (30 min)	17345.5	0.350
Perfusate 7 (35 min)	20,211	0.578
Perfusate 8 (40 min)	*21,492*	*0.875*
Perfusate 9 (45 min)	*22537.5*	*0.833*
Perfusate 10^*^ (50 min)	*12540.5*	*0.500*
Bile 1	21,604	0.375
Bile 2	31,988	0.152
Liver 1	44,440	0.477
Liver 2	29,041	0.727

No significant correlation between the FI of the samples and other endpoints, such as morbidity Clavien-Dindo ≥ 3, ICU stay or hospital stay could be evaluated. A significant influence of donor risk factors, such as CIT or donor transaminases on ICG kinetics could not be observed.

## Discussion

This pilot study aimed to evaluate the diagnostic potential of fluorescence-based ICG measurement during HOPE to predict graft viability. Due to the lack of long- term follow- up data, EAD was used as a surrogate endpoint, consistent with the approaches of Schlegel et al. and Rauter et al., who also used EAD to assess early graft performance during HOPE [20]. Schlegel et al. demonstrated the use of FMN as viability marker for hypothermic perfused livers. Mitochondrial injury represents a central mechanism of ischemia- reperfusion damage and has been extensively studied in the HOPE context. During HOPE, the restoration of oxygen supply allows reactivation of the mitochondrial respiratory chain and mitochondrial dysfunction can be assessed by the release of FMN, which reflects complex I injury. In contrast, ICG kinetics may provide complementary information on hepatocellular function. Following uptake into hepatocytes via OATP, ICG is excreted into the bile through MRP2. Impaired hepatocellular energy metabolism or transporter activity may therefore alter ICG uptake and clearance during machine perfusion [6–8].

Our findings demonstrate a time- dependent pattern of ICG metabolism during machine perfusion. Specifically, perfusate samples collected at 40–45 min post- ICG injection (perfusate 8, 9) exhibited the highest predictive value for EAD (AUC = 0.875 and 0.889, respectively). This supports the concept that viable grafts actively take up and clear ICG within this time frame, while dysfunctional grafts may show delayed clearance- a dynamic which is already described in the context of ICG- based liver function assessment in hepatobiliary surgery [9, 12]. Earlier perfusate samples (perfusate 1–6) showed no, respectively moderate discriminatory capacity, indicating the necessity of allowing sufficient time for hepatocellular uptake during hypothermic perfusion. This finding is in line with the observation that, during hypothermic oxygenated machine perfusion, hepatocyte metabolism is still detectable- albeit significantly reduced compared to physiological normothermia [20].

Although ROC/AUC analyses suggested a potential discriminatory association within this cohort, the limited sample size precludes robust determination of clinically applicable cutoff values, and these findings should therefore be considered exploratory pending external validation in larger studies.

Liver tissue fluorescence after reperfusion (Liver 2) also showed discriminatory capacity (AUC 0.886), reinforcing the relevance of mitochondrial and hepatocellular integrity for early graft function [9]. In contrast, fluorescence intensity from bile samples- particularly after reperfusion- showed a clear tendency, especially in contrast to the liver sample after reperfusion but ultimately a poor discrimination (AUC 0.364). These inconsistencies are likely due to not optimal sample timing, possible dilution with perfusate, and contamination with blood during sampling after reperfusion.

In this exploratory pilot study, perfusate fluorescence measurements during HOPE were associated with EAD in this cohort. These findings suggest that fluorescence- based assessment during machine perfusion may provide functional information on graft quality.

The observed peak in discriminatory performance at 40–45 min after ICG administration may reflect the phase of maximal hepatocellular uptake during HOPE. Following injection into the portal perfusion circuit, ICG first may distribute within the perfusate and sinusoidal compartment before being actively taken up by hepatocytes via organ anion transporting polypeptides (OATP1B1/1B3) [10–12]. Differences in hepatocellular function between grafts may therefore become most apparent once cellular uptake predominates. Given the growing clinical use of HOPE due to its simplicity, safety and strong protective effects against ischemia- reperfusion injury (IRI), the integration of ICG- based functional evaluation could further enhance graft assessment- especially for extended criteria donors (ECD), where decision- making remains complex [5, 7–9].

Exploratory correlation analyses demonstrated a positive association between perfusate fluorescence intensity at 40 and 45 min and postoperative AST levels. AST is widely regarded as marker of hepatocellular damage. In contrast, no significant correlations were observed with INR or bilirubin. These findings support the concept that fluorescence- based assessment during HOPE may provide functional information on hepatocellular integrity before transplantation. Given the limited sample size of this pilot study, these findings should be interpreted as exploratory and require validation in larger cohorts.

This study has several limitations that must be acknowledged and should be considered an exploratory proof-of-concept investigation. First, the sample size is relatively small, reflecting the pilot nature of this investigation, which may limit the generalizability of the reported findings. As a result, the statistical power to validate proposed cutoff values or detect subtle differences between the groups is reduced and the findings of this study should be interpreted as hypothesis- generating. Due to the limited cohort size and the small number of EAD events, multivariable adjustment for potential risk factors is not statistically feasible. Consequently, residual confounding cannot be excluded. Validation in larger, independent cohorts will be necessary to confirm the predictive value of perfusate fluorescence intensity during HOPE. EAD was chosen as a surrogate endpoint for graft viability due to the lack of an adequate follow-up period. It remains an imperfect instrument that does not fully capture long-term graft function or clinical outcome. Accordingly, the present study does not establish prediction of long- term graft survival, graft loss, or patient survival, but only demonstrates an association between perfusate fluorescence measurements during HOPE and the occurrence of early allograft dysfunction.

Additionally timing and standardization of bile sampling proved to be challenging, particularly during the early phase of this trial, potentially affecting reliability of the fluorescence measurements in the bile and due to missing data in later perfusate point perfusate 10, some time dependent analyses had to be omitted. Finally this study was conducted at a single center. Further studies with larger cohorts, optimized standardized sampling procedures and long-term follow- up are needed to validate the results and assess their clinical applicability.

Another limitation relates to the exploratory assessment of multiple perfusate time points, tissue samples and bile samples. Statistical significance was observed only at 40–45- minute perfusate measurements. Given the number of comparisons performed and the absence of adjustment for multiple testing, these findings may partly reflect chance rather than a true biologically optimal sampling window. Future studies with larger cohorts and predefined sampling time points are required to validate whether this interval represents the optimal assessment window.

The ROC analyses were based on five EAD events, therefore the reported AUC estimates should be interpreted with caution, as discrimination metrics derived from such small event numbers are prone to instability and may overestimate predictive performance. Consequently, these results should be interpreted as exploratory and hypothesis- generating until confirmed in larger prospective cohorts.

## Data Availability

The datasets generated and analyzed during the current study are not publicly available due to patient privacy and institutional data protection regulations but are available from the corresponding author on reasonable request.
